# The non-random landscape of somatically-acquired uniparental disomy in cancer

**DOI:** 10.18632/oncotarget.26987

**Published:** 2019-06-18

**Authors:** Pau Erola, Keyvan Torabi, Rosa Miró, Jordi Camps

**Affiliations:** CRUK Integrative Cancer Epidemiology Programme, MRC Integrative Epidemiology Unit, University of Bristol, Bristol, BS8 2BN, UK; Gastrointestinal and Pancreatic Oncology Group, Institut D’Investigacions Biomèdiques August Pi i Sunyer (IDIBAPS), Centro de Investigación Biomédica en Red de Enfermedades Hepáticas y Digestivas (CIBEREHD), Barcelona, Catalonia, 08036, Spain; Unitat de Biologia Cel·lular i Genètica Mèdica, Departament de Biologia Cel·lular, Fisiologia i Immunologia, Facultat de Medicina, Universitat Autònoma de Barcelona, Bellaterra, Catalonia, 08193, Spain; Gastrointestinal and Pancreatic Oncology Group, Institut D’Investigacions Biomèdiques August Pi i Sunyer (IDIBAPS), Centro de Investigación Biomédica en Red de Enfermedades Hepáticas y Digestivas (CIBEREHD), Barcelona, Catalonia, 08036, Spain

**Keywords:** somatically-acquired uniparental disomy, uniparental disomy, copy-neutral loss of heterozygosity, copy number alterations, cancer

Uniparental disomy (UPD) was first described in 1980 by Engel and was defined as the occurrence of inheriting a pair of homologous chromosomes from the same parental origin. Such event, also known as constitutional UPD, is caused by segregation errors mostly in meiosis and leads to the development of several genetic disorders through the gain or loss of imprinted regions, or the presence of two identical copies of an abnormal gene [1]. The great advance in molecular genetics in the last two decades, especially with the development of single nucleotide polymorphism (SNP) arrays and next-generation sequencing, has provided the opportunity to systematically identify regions of somatic UPD in cancer, commonly known as somatically-acquired UPD (aUPD) or copy-neutral loss of heterozygosity (cnLOH) [2, 3].

The detection of allele-specific copy number aberrations in tumor cells relies strongly on bioinformatic tools that were initially developed for SNP array technologies. These methods are strongly dependent on each technology-associated signal and noise characteristics, tumor ploidy, the cellularity of the samples, variant allele bias in heterozygous loci, and the tumor type [4]. Moreover, allele-specific copy number analysis requires heterozygous SNP markers, which represent on average less than 1/3 of the covered positions. Altogether, it is common that these models fail to analyze many samples or return low confidence predictions. More recently, some tools have been proposed for next-generation sequencing data. While whole-exome sequencing lacks an even coverage of the genome and heterozygous positions, leading to a potential lower accuracy, whole-genome sequencing data promise highly precise estimates of allele-specific copy number profiles and a better integration with mutational status and epigenetic marks.

**Figure 1:**
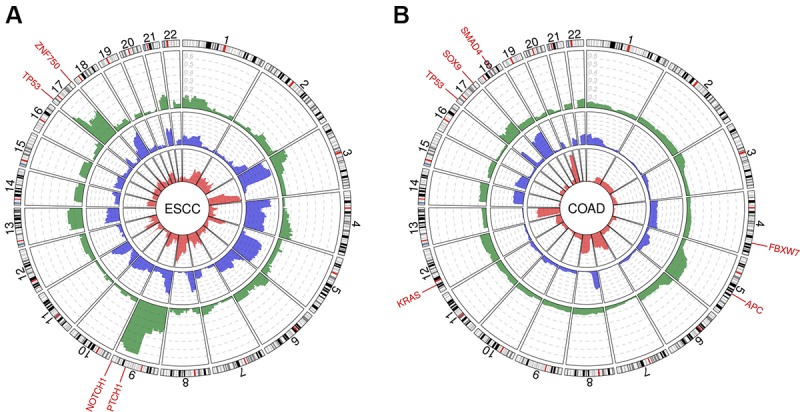
Circos plots depicting genomic profiles of aUPDs (green), copy number losses (blue) and gains (red) in (**A**) esophageal squamous cell carcinomas (ESCC) and (**B**) colon adenocarcinomas (COAD). Data was obtained from The Cancer Genome Atlas (TCGA) through the NCI Genomics Data Commons (GDC) portal. In the outer ring, the most frequently mutated genes involved in aUPD events are indicated for each tumor type. (Adapted from Torabi *et al*., 2019).

Recent reports have shown that aUPD events are non-randomly distributed across cancer types, pointing out common genomic profiles of aUPD in a tumor-type specific manner, which typically coincide with regions of genomic losses, both in solid tumors and hematological malignancies [5, 6]. In addition, the identification of cancer-specific minimal regions of aUPD has led to the discovery of inactivating mutations in cancer-related genes, which might increase the functional relevance of such events in the development of cancer. In fact, several studies have provided evidence that aUPD could act as the “second hit” in the Knudson hypothesis and inactivate tumor suppressor genes. Examples of this phenomenon are the inactivation of well-known tumor suppressor genes such as *APC* in colorectal cancer, *RB1* in retinoblastoma, *ARID1A* in colorectal and stomach carcinoma, and inactivation of *TP53* in several cancer types, including glioblastoma, diffuse large B cell lymphoma, and colorectal carcinoma, among others [7]. Moreover, some studies have identified aUPD in colorectal pre-malignant adenomas, mostly affecting *APC* and *SMAD4*, two tumor suppressor genes known to be involved in early colorectal carcinogenesis [8]. Likewise, aUPD also occurs in genomic regions including proto-oncogenes such as *KRAS* at 12p, indicating that aUPD also plays a crucial role in the activation of oncogenes (Figure 1).

The mechanisms by which aUPD is generated remain elusive. aUPD events are either caused by chromosome segregation errors in mitosis, leading to whole-chromosome aUPD, or by mitotic recombination, which acts as a repairing double strand break mechanism and might lead to segmental aUPD. In fact, segmental aUPD is commonly identified in tumors with impaired chromosome repair mechanisms, such as *MUTYH*-associated polyposis colon carcinomas [9]. It might be plausible to rationalize that mitotic recombination-associated segmental aUPD might imply two features: (i) genomic sites of high sequence homology, which coincide with sites of meiotic recombination as seen for chromosome 5q breakpoints [10], and (ii) a physical proximity of homologous chromosomes that would increase the chances of mitotic recombination events, especially in aneuploid tumors where the higher-order genome organization might be altered.

Several questions remain to be answered to further understand the role of aUPD in cancer. The higher prevalence of aUPD in certain chromosomal regions compared with the frequency of genomic losses (e.g., chromosome 5q in colorectal cancer), suggests that tumor cells need to preserve such chromosomal regions in two or more copies in order to maintain cellular fitness, most likely due to the presence of haploinsufficient genes or genomic regions under negative selection in a tissue-specific manner. At the functional level, the transcriptional consequences of aUPD, as well as the gene dysregulation due to the disruption of imprinted gene clusters during the carcinogenesis, prevail largely unknown. Lastly, the association between aUPD and disease outcome in large cohorts is rather limited. Therefore, additional studies are required to elucidate the prognostic potential of recurrent aUPD events in cancer.

In summary, although we cannot exclude that a second sequence mutation, epigenetic modifications or DNA conformational changes might lead to loss-of-function of tumor suppressor genes or activation of proto-oncogenes, a substantial amount of literature suggests that aUPD represents an unquestionable event to achieve a “second hit” alteration during tumorigenesis and its identification might be a useful resource in early detection and targeted therapy.
